# Effect of the Ca_2_Mg_6_Zn_3_ Phase on the Corrosion Behavior of Biodegradable Mg-4.0Zn-0.2Mn-*x*Ca Alloys in Hank’s Solution

**DOI:** 10.3390/ma15062079

**Published:** 2022-03-11

**Authors:** Junjian Fu, Wenbo Du, Ke Liu, Xian Du, Chenchen Zhao, Hongxing Liang, Adil Mansoor, Shubo Li, Zhaohui Wang

**Affiliations:** Faculty of Materials and Manufacturing, Beijing University of Technology, Beijing 100124, China; fujunjian@emails.bjut.edu.cn (J.F.); duxian@bjut.edu.cn (X.D.); zhaochenchen@bjut.edu.cn (C.Z.); hongxingliang314@gmail.com (H.L.); adilmansoor_786@outlook.com (A.M.); lishubo@bjut.edu.cn (S.L.); wangzhaohui@bjut.edu.cn (Z.W.)

**Keywords:** Mg-Zn-Mn-Ca, Ca_2_Mg_6_Zn_3_ phase, corrosion behaviors, SKPFM

## Abstract

The effect of the Ca_2_Mg_6_Zn_3_ phase on the corrosion behavior of biodegradable Mg-4.0Zn-0.2Mn-*x*Ca (ZM-xCa, x = 0.1, 0.3, 0.5 and 1.0 wt.%) alloys in Hank’s solution was investigated with respect to phase spacing, morphology, distribution and volume fraction. With the increase in Ca addition, the volume fraction of the Ca_2_Mg_6_Zn_3_ phase increased from 2.5% to 7.6%, while its spacing declined monotonically from 43 μm to 30 μm. The Volta potentials of secondary phases relative to the Mg matrix were measured by using scanning kelvin probe force microscopy (SKPFM). The results show that the Volta potential of the intragranular spherical Ca_2_Mg_6_Zn_3_ phase (+109 mV) was higher than that of the dendritic Ca_2_Mg_6_Zn_3_ phase (+80 mV). It is suggested that the Ca_2_Mg_6_Zn_3_ acted as a cathode to accelerate the corrosion process due to the micro-galvanic effect. The corrosion preferred to occur around the spherical Ca_2_Mg_6_Zn_3_ phase at the early stage and developed into the intragranular region. The corrosion rate increased slightly with increasing Ca content from 0.1 wt.% to 0.5 wt.% because of the enhanced micro-galvanic corrosion effect. The decrease in the phase spacing and sharp increase in the secondary phase content resulted in a dramatic increase in the corrosion rate of the ZM-1.0Ca alloy.

## 1. Introduction

Magnesium (Mg) alloys have attracted great attention as promising biodegradable materials for orthopedic implants and cardiovascular interventional devices [1,2,3,4,5]. Compared with traditional metallic biomaterials, such as stainless steels and titanium alloys, Mg alloys reduce the stress-shielding risk [6]. Mg alloys usually degrade within a few weeks, and the produced Mg^2+^ ions can be advantageous for bone healing without toxicity [7]. However, the uncontrolled corrosion of Mg alloys may lead to unexpected mechanical failure before tissue recovery. This is a major problem for Mg alloys being used as clinical implants in a physiological environment with a high chloride and/or pH of 7.4–7.6 [7]. Therefore, controlling the corrosion of Mg alloys is becoming an urgent problem.

Alloying is one of the most effective methods to improve corrosion resistance and sustain the mechanical properties of Mg alloys [8,9]. Aluminum (Al) and rare earth elements are usually selected to modify the degradation rate of Mg alloys, but their adverse effects on biocompatibility with the human body have to be fully considered. For example, excessive Al usually causes nerve toxicity and restrains human body growth [10,11,12,13,14], while rare earth elements increase the risk of thrombosis [3]. In addition, alloying may result in precipitation of secondary phases and typically accelerate galvanic corrosion of Mg alloys, leading to the consequence of local corrosion and collapse of Mg implants. Thus, it is necessary to design and control secondary phases by choosing reasonable alloying elements, as well as heat treatments, to reduce their impacts on the corrosion rate of Mg alloys.

Manganese (Mn) is a beneficial element for improving the corrosion resistance of Mg alloys [15,16,17,18]. Studies have shown that Mn addition resulted in forming a MnO_2_ film on the surface of Mg alloys, inhibiting the permeation of chloride ions and improving corrosion resistance [17,18]. Zinc (Zn) is one of the most essential elements for the physiological functions of the human body [2], but Zn accelerates the corrosion rate because of forming the Mg_x_Zn_y_ phase [19,20,21]. As reported by Song [22], the Mg_x_Zn_y_ phase acted as a micro-cathode and accelerated corrosion as its volume fraction increased. Calcium (Ca) is an indispensable element of human bone, which not only accelerates bone healing [23] but also enhances corrosion resistance and mechanical performance of the implant. When Ca is added to Mg-Zn alloys, the Mg_2_Ca and Ca_2_Mg_6_Zn_3_ phases are usually formed according to the atomic ratio of Zn/Ca [24,25,26]. Although previous studies have indicated that the Ca_2_Mg_6_Zn_3_ phase has dominating effects on the corrosion rate of Mg-Zn-Ca alloys [27,28,29,30], its detailed corrosion behavior has not been entirely understood. In this work, we have focused on the effects of the Ca_2_Mg_6_Zn_3_ phase with respect to its morphology, distribution and volume fractions on the corrosion behavior of Mg-4.0Zn-0.2Mn-*x*Ca alloys in Hank’s simulate solution, a solution similar to human body fluid, which is generally used for in vitro corrosion experiments. Its composition is given in Section 2.4. The corrosion mechanism is discussed.

## 2. Materials and Methods

### 2.1. Material Fabrication

The materials used in this work were cast in the laboratory. The as-cast Mg-4.0Zn-0.2Mn-*x*Ca alloys (denoted as ZM-xCa alloys, x = 0.1, 0.3, 0.5 and 1.0 wt.%) were prepared using high-purity Mg (99.95 wt.%), high-purity Zn (99.99 wt.%), Mg-Mn and Mg-Ca master alloys procured from Hunan Research and Institute of Rare Earth and Material. Melting (Changsha, China) was conducted in an electrical resistance furnace under a mixed gas of N_2_ and SF_6_ (the ratio of N_2_ and SF_6_ was 100:1). The melt was kept at 750 °C for 20 min, then poured into a steel mold (preheated at 200 °C) at 720 °C and cooled to room temperature. The actual chemical compositions of the ZM-xCa alloys were examined by X-ray fluorescence (XRF, Magix-PW2403 with a test resolution of 0.0001), as shown in Table 1.

### 2.2. Microstructural Characterization

Microstructure observation was conducted using an optical microscope (AXIO IMAGER A2M) and a scanning electron microscope (SEM, HITACHI S3400N, HITACHI, Tokoy, Japan) equipped with energy dispersive X-ray spectroscopy (EDS). The nominal distance between secondary phases (phase spacing) and the volume fraction of precipitates were determined by Image-Pro Plus 6.0 software (Media Cybernetics, Houston, TX, USA), and 10 images were selected for data statistics at least. The samples for optical microscope observation were ground up to 5000# SiC grit papers (Federation of European Producers of Abrasives, FEPA standard) and polished with 0.5 μm diamond paste, etched by 5% HNO_3_/C_2_H_6_O solution for 10~20 s. X-ray diffraction (XRD, D/MAX-3C, Rigaku, Tokyo, Japan) with Cu Kα radiation was used to analyze phases with a step of 0.02° in the scanning range of 10~90° at room temperature. The microstructure features of secondary phases were characterized by transmission electron microscopy (TEM, JEM-2100, JEOL, Tokyo, Japan) equipped with an Oxford energy spectrum system (EDX can give semi-quantitative analysis, Oxford Instruments, Abingdon, UK) at an accelerating voltage of 200 kV. The dwelling time was 100 ms, and the anode voltage was 15 mV, with a current of 10 mA. Three-dimensional (3D) distribution of secondary phases was analyzed by X-ray Microscope (XRM, Xradia 520 Versa, ZEISS, Oberkochen, Baden-Württemberg, Germany) with a high effective pixel size of 1.5 μm at a voltage of 80 kV and a power of 7 W. The specimen (cylindrical shape) with dimensions of Φ1.8 mm × 25 mm was prepared for the 3D morphology test. The Object Research System (ORS) Visual software (Object Research System, Montreal, QC, Canada) was used to analyze the experimental data obtained by X-ray Microscope to reconstruct the 3D tomography image of the secondary phase.

### 2.3. Local Volta Potential Measurement

The potentials (relative nobility potential) of secondary phases were measured by scanning kelvin probe force microscopy (SKPFM, Bruker Icon, Bruker, Billerica, MA, USA) in the tapping mode at room temperature (~25 °C) and a relative humidity of ~50%. A magnetic etched silicon probe was used to measure the relative Volta potential, with a bias potential of 5 V applied to the sample. The tip height is 100 nm, pixel resolution is 20 nm, and scan rate is 0.5 Hz. Prior to the SKPFM test, the samples were polished with diamond paste and alcohol. The samples were vacuum packed immediately after drying with cold air. The Volta potential differences were analyzed using NanoScope Analysis 2.0 software (Bruker, Billerica, MA, USA).

### 2.4. In Vitro Immersion Test

The immersion test was carried out in Hank’s physiological solution (8.00 g/L NaCl, 0.40 g/L KCl, 0.14 g/L CaCl_2_, 0.35 g/L NaHCO_3_, 0.1 g/L MgCl_2_·6H_2_O, 0.06 g/L MgSO_4_·7H_2_O, 0.06 g/L KH_2_PO_4_ and 0.06 g/L Na_2_HPO_4_·12H_2_O, pH = 7.4) at 37 ± 0.4 °C with different periods. The samples (10 × 10 × 10 mm^3^) used for corrosion studies were cut from the cast block, and then ground up to 2000# SiC grit papers. Subsequently, the samples were cleaned ultrasonically with acetone and ethanol solutions, respectively. The ratio of sample surface area to solution volume was 1 cm^2^:150 mL and the solution was renewed every two days. After immersion, the samples for SEM were ultrasonically cleaned with 200 g/L CrO_3_ + 10 g/L AgNO_3_ solution for 10 min to remove the surface corrosion products. The average corrosion rate (estimated by weight loss, *Vc*) was calculated according to the following equation [31,32]:

(1) $$V_{C}=\frac{{3650\;\times \;(W}_{o}\;{-\;W}_{t})}{\mathrm{DAT}}$$

where, *V_C_* is the corrosion rate (mm/y), *W_o_* is the sample weight before immersion (g), *W_t_* is the sample weight after removing corrosion products (g), *D* is the density (g/cm^3^), *A* is the sample surface area exposed to the solution (cm^2^), and *T* is the immersion time (d).

### 2.5. Electrochemical Measurement

The samples were sealed in epoxy resin to expose only one surface (1 cm^2^) for electrochemical measurement. The measurement was conducted by an electrochemical workstation (Autolab, Metrohm, Herisau, Appenzell Ausserrhoden, Switzerland) with a three-electrode system, in which the saturated calomel electrode (SCE), the platinum mesh and the sample were used as reference electrode, counter electrode and working electrode, respectively. The potentiodynamic polarization tests were performed in Hank’s solution (about 200 mL) at a constant scanning rate of 0.5 mv/s, and the voltage range to open circuit potential (OCP) was ±300 mv, and the OCP monitored time was 120 s. The electrochemical parameters, such as corrosion potential (*E_corr_*) and corrosion current density (*i_corr_*) were obtained by Tafel extrapolation. The electrochemical impedance spectroscopy (EIS) measurement was carried out in a frequency range from 100 kHz to 100 mHz with a perturbation of 10 mV, and the EIS data (Frequency, Z’ and Z’’, about 220 data points) were fitted using ZView3.1 software (Scribner Associates, Southern Pines, NC, USA). All measurements were repeated at least three times to ensure the reproducibility of the results.

## 3. Results

### 3.1. Microstructure of ZM-xCa Alloys

**Figure 1:**
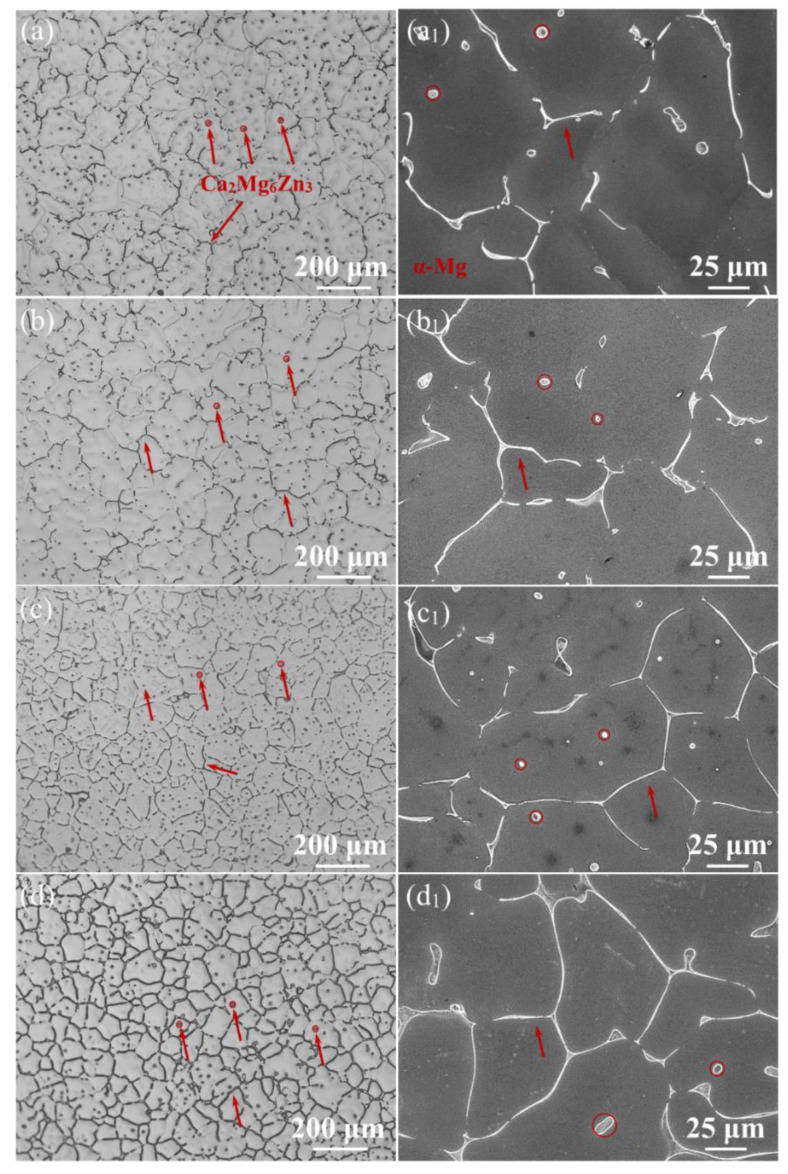
OM (**a**–**d**) and SEM (**a_1_**–**d_1_**) micrographs of the as-cast ZM-0.1Ca (**a**,**a_1_**), ZM-0.3Ca (**b**,**b_1_**), ZM-0.5Ca (**c**,**c_1_**) and ZM-1.0Ca (**d**,**d_1_**) alloys.

Figure 1 shows the OM (a–d) and SEM (a_1_–d_1_) micrographs of the ZM-xCa alloys. As shown in Figure 1, the dendritic and spherical phases are mainly distributed along grain boundaries and within grains, respectively. Although the distribution of the secondary phases hardly changed, the grain size decreased with an increase in the Ca amount. Table 2 lists the microstructure characteristics of the ZM-xCa alloys.

This indicates that the total volume fraction of the secondary phases increased from 2.5% to 7.6%, while spacing declined from 43 μm to 30 μm when the added Ca amount increased from 0.1 wt.% to 1.0 wt.%.

**Figure 2:**
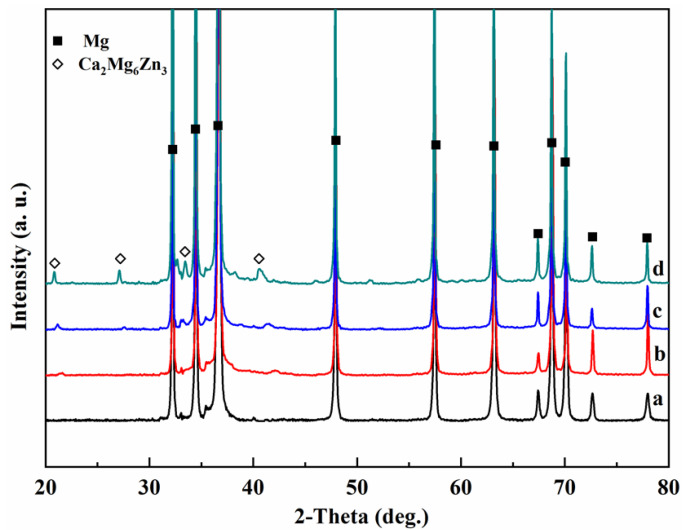
XRD patterns of ZM-xCa alloys: (**a**) ZM-0.1Ca, (**b**) ZM-0.3Ca, (**c**) ZM-0.5Ca and (**d**) ZM-1.0Ca.

**Figure 3:**
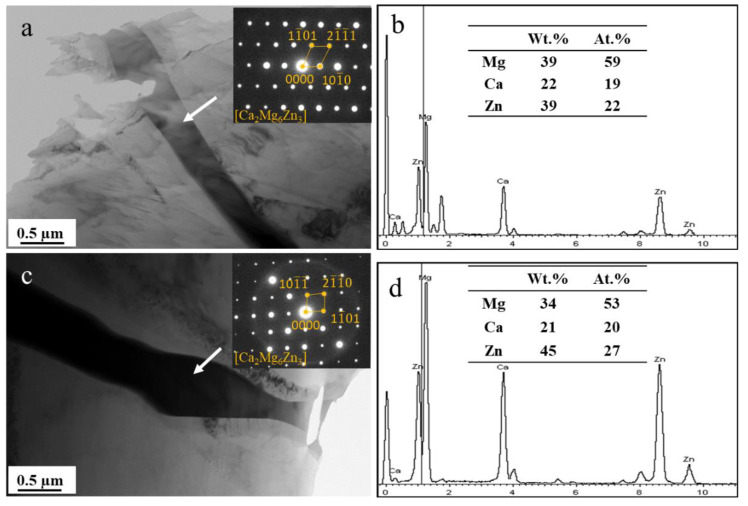
TEM images and EDS results of the secondary phases: (**a**,**b**) ZM-0.3Ca and (**c**,**d**) ZM-0.5Ca alloys.

Figure 2 shows the XRD patterns of the ZM-xCa alloys. Only α-Mg and Ca_2_Mg_6_Zn_3_ peaks are present in all ZM-xCa alloys. In addition, the diffraction intensity of the Ca_2_Mg_6_Zn_3_ peak increased with an increase in the Ca amount, indicating that Ca promoted the eutectic precipitation of the Ca_2_Mg_6_Zn_3_ phase. Figure 3 shows the TEM images and EDS results of the ZM-0.3Ca and ZM-0.5Ca alloys. The phases in these two alloys were located at grain boundaries and had a strip morphology. According to the EDS results, these secondary phases are inferred to be Ca_2_Mg_6_Zn_3_, showing a hexagonal crystal structure [33,34,35,36].

### 3.2. Electrochemical Properties of the ZM-xCa Alloys

**Figure 4:**
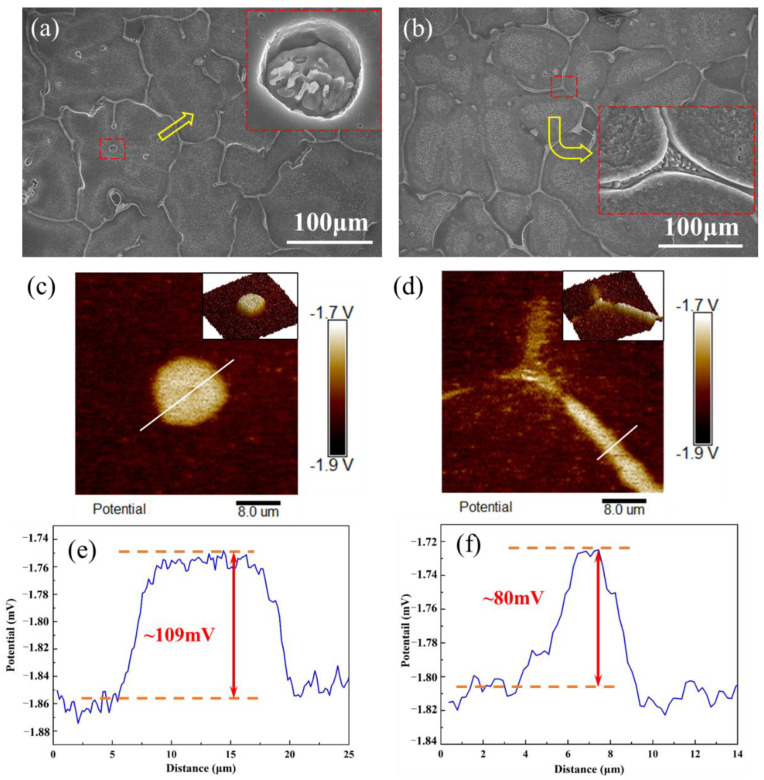
SEM images and SKPFM analysis of the ZM-0.3Ca alloy: (**a**) SEM images of the spherical secondary phase and (**b**) the dendritic secondary phase; (**c**) SKPFM maps of the spherical secondary phase and (**d**) the dendritic secondary phase; (**e**) the Volta potential profile of line as indicated in (**c**); (**f**) the Volta potential profile of line as indicated in (**d**).

Two typical Ca_2_Mg_6_Zn_3_ phases, namely the spherical (Figure 4a) and dendritic (Figure 4b) phases in the ZM-0.3Ca alloy, were chosen to conduct SKPFM analysis. The Volta potential differences between Ca_2_Mg_6_Zn_3_ phases and Mg matrix are revealed in Figure 4c–f.

From Figure 4c,d, it can be determined whether the spherical or the dendritic phase is brighter than the Mg matrix. The SKPFM work function mode supports the idea that the light color demonstrates more positive potential than the dark one, i.e., the light color represents the cathodic area, whereas the dark one stands for anodic one [37,38]. According to the line-profile analyses shown in Figure 4e,f, the spherical phase exhibited a relatively higher Volta potential difference (about +109 mV) than the dendritic phase (about +80 mV). These results imply that the spherical phase has a stronger acceleration effect than micro-galvanic corrosion.

**Figure 5:**
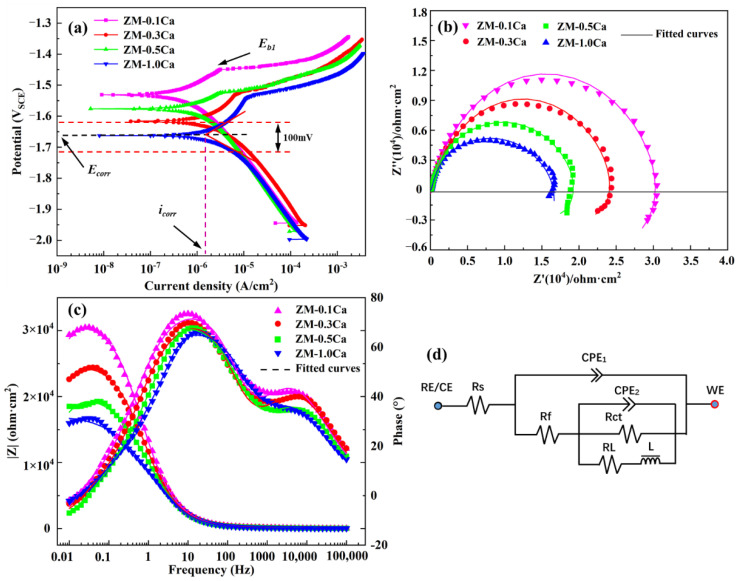
Potentiodynamic polarization curves (**a**) and electrochemical impedance spectra of the ZM-xCa alloys: (**b**) Nyquist plots, (**c**) Bode impedance and Bode phase angle plots, and (**d**) Equivalent circuit.

The potentiodynamic polarization curves of the ZM-xCa alloys in Hank’s solution are presented in Figure 5a. The values of electrochemical parameters, i.e., corrosion potential (*E_corr_*), corrosion current density (*i_corr_*) and breakdown potential (*E_b_*) are summarized in Table 3. It can be found that the *E_corr_* moved in the negative direction, but the *i_corr_* consistently increased with an increase in the Ca amount, and the ZM-0.1Ca alloy displayed the lowest *i_corr_* value of 5.90 × 10^−7^A/cm^2^. In the potentiodynamic polarization test, the corrosion potential is a thermodynamic parameter that reflects the probability of the corrosion tendency, while the corrosion current density is a kinetic parameter that signifies the corrosion rate [39]. The most positive corrosion potential, as well as the lowest corrosion current density of the ZM-0.1Ca alloy, indicates that it has the best corrosion resistant property. Moreover, the ZM-0.1Ca alloy has the most positive breakdown potential (Eb) of −1.450 V, which indicates that the corrosion reaction is more difficult to proceed because of the formed corrosion film [40].

Figure 5b–d indicates the EIS results of the ZM-xCa alloys. As shown in Figure 5b, the Nyquist plots of the four alloys are composed of capacitance loops at high frequency and an inductive loop at low frequency. All of the Nyquist plots are similar in shape except for the diameter of the loop. This result demonstrates that the corrosion mechanism of the four alloys is similar, but their corrosion rates are different. According to the Nyquist plots, the corrosion rate ranks as ZM-0.1Ca ˂ ZM-0.3Ca ˂ ZM-0.5Ca ˂ ZM-1.0Ca. In addition, the inductive loop at a low frequency implies a breakdown of the protective corrosion-products film [41]. Figure 5c shows the impedance modulus (|Z|), as well as the Bode phase angle plots of the ZM-xCa alloys. The largest impedance modulus (|Z|) of the ZM-0.1Ca alloy represents the best corrosion protection resulting from the corrosion-product film. The maximum crest of the Bode phase angle plot also reflects the difficulty of charge transfer of the ZM-0.1Ca alloy. Figure 5d indicates the fitted equivalent circuit using the EIS data. *R_s_*, *R_f_* and *R_ct_* are solution resistance, film resistance and charge transfer resistance, respectively. *CPE_1_* represents the constant phase element of corrosion product film, *CPE_2_* represents the double layer capacitance. *L* and *R_L_* are the inductance and inductance resistance. The detailed fitting results are listed in Table 4.

The polarization resistance (*R_p_*), which is inversely proportional to the corrosion rate, is an important parameter for evaluating the corrosion performance of an alloy. Its value was calculated using the following equation:

(2) $$R_{P}{=R}_{S\;}{+R}_{f}+\frac{R_{\mathrm{ct}}R_{L}}{R_{\mathrm{ct}}{+R}_{L}}$$

The calculated *R_p_* of the ZM-xCa alloys is also listed in Table 4. The largest *R_p_* of the ZM-0.1Ca alloy demonstrated its best corrosion resistance, which is consistent with the polarization result. 

### 3.3. Corrosion Behaviors of ZM-xCa Alloys

**Figure 6:**
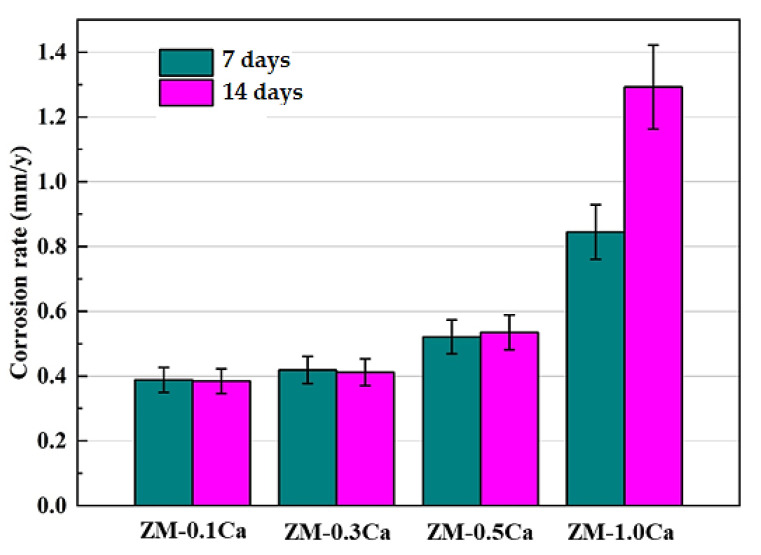
Corrosion rates of the ZM-xCa alloys obtained by weight loss after immersion in Hank’s solution for different times.

Figure 6 shows the corrosion rates of the ZM-xCa alloys by weight loss after immersion in Hank’s solution for 7 days and 14 days, respectively.

The corrosion rate increases with an increase in the added Ca amount for neither 7-day nor 14-day immersion. The corrosion rate increased slowly when the added Ca amount was less than 0.5 wt.%; however, it sharply increased for the ZM-1.0Ca alloy. In particular, the 14-day corrosion rate of the ZM-1.0Ca alloy is about three times that of the ZM-0.1Ca alloy. These results demonstrate that excessive Ca addition destructs the corrosion resistance of the ZM-xCa alloys.

**Figure 7:**
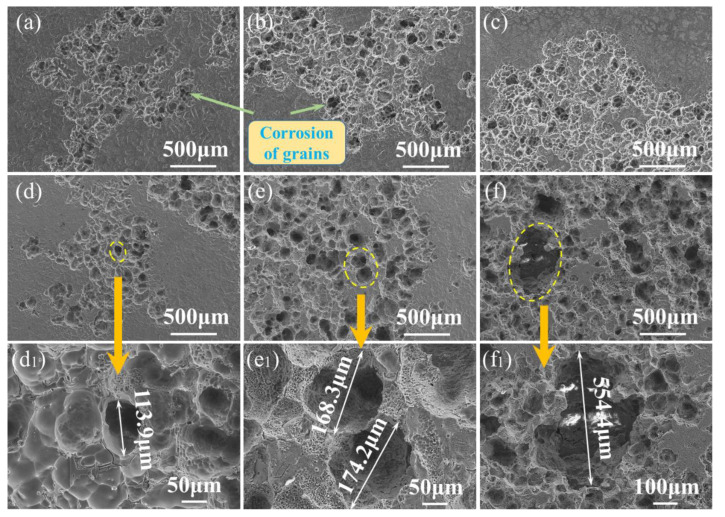
Surface morphologies after removal of the corrosion products with various immersion times in Hank’s solution of the ZM-xCa alloys: (**a**–**c**) ZM-0.1Ca, ZM-0.3Ca and ZM-0.5Ca alloys immersed for 7-day; (**d**,**d_1_**), (**e**,**e_1_**) and (**f**,**f_1_**) ZM-1.0Ca alloy immersed for 3-day, 7-day and 14-day, respectively.

The surface morphologies after removal of corrosion products of the ZM-xCa alloys with various immersion times are displayed in Figure 7. After 7-day immersion, a shallow corrosion area extended with an increase in the added Ca amount, as shown in Figure 7a–c,e. On the other hand, corrosion morphology also changes significantly with corrosion time. In the case of the ZM-1.0Ca alloy, plenty of tiny corrosion pits appeared after 3-day immersion (Figure 7d), and an almost whole corroded morphology was presented after 7-day immersion (Figure 7e). The corrosion pits became corrosion cavities with an extension of the corrosion time. The corrosion cavity became deeper and wider and reached about 554 μm after 14-day immersion (Figure 7d_1_–f_1_).

## 4. Discussion

**Figure 8:**
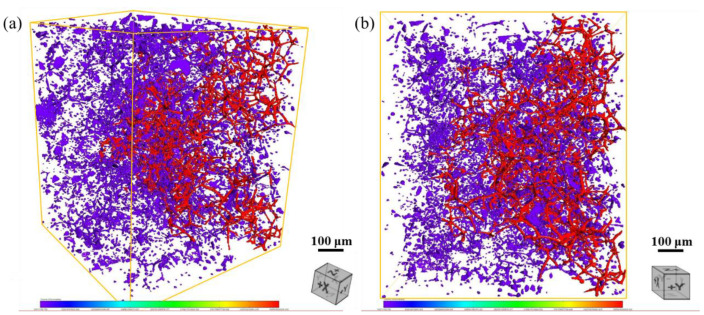
Three-dimensional topographical characteristics of the Ca_2_Mg_6_Zn_3_ secondary phase in the ZM-0.3Ca alloy: (**a**) perspective view, (**b**) sectional view of the perpendicular Y axle.

The Ca_2_Mg_6_Zn_3_ phase plays a significant role in the corrosion process of magnesium alloys [42,43]. In the present study, the corrosion behavior of the ZM-xCa alloys is investigated by altering the Ca_2_Mg_6_Zn_3_ phase morphology, distribution and volume fraction. Figure 8 shows the 3D topographical characteristics of the Ca_2_Mg_6_Zn_3_ phase in the ZM-0.3Ca alloy, giving an intuitive distribution of the secondary phase. It was found that there are two kinds of morphologies of the Ca_2_Mg_6_Zn_3_ phase, one spherical and the other dendritic. In addition, the dendritic (marked with red color) Ca_2_Mg_6_Zn_3_ phase precipitated at grain boundaries and connected with each other, forming a continuous network, while the spherical (marked with violet color) Ca_2_Mg_6_Zn_3_ phase was distributed within grains. These results are consistent with the optical images shown in Figure 1.

**Figure 9:**
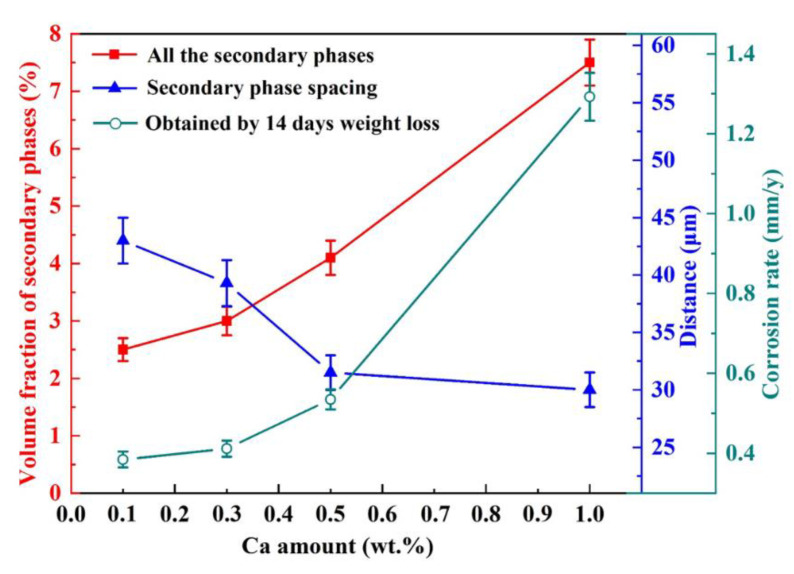
Relationship between corrosion rate, secondary phase spacing and volume fraction of the ZM-xCa alloys.

The Volta potential in air is widely used in the corrosion field to assess the corrosion tendency of Mg alloys [42,43,44]. According to the SKPFM results shown in Figure 4, the Volta potential of either the dendritic or the spherical Ca_2_Mg_6_Zn_3_ phase is higher than that of the Mg matrix; hence, not only the dendritic but also the spherical Ca_2_Mg_6_Zn_3_ phase acts as a cathode, while the Mg matrix acts as an anode in micro-galvanic corrosion. As a result, the Mg matrix dissolves preferentially during the immersion period. Therefore, the total amount of the Ca_2_Mg_6_Zn_3_ phase, as well as its phase spacing, plays a controlling role in the corrosion process; that is, the higher the volume fraction and/or the smaller the phase spacing, the higher the corrosion rate. Figure 9 shows the relationship between the corrosion rate, the volume fraction and the spacing of the secondary phase in the ZM-xCa alloys. With the increase in the volume fraction and decrease in the spacing of the secondary phase, the corrosion rate of the ZM-xCa alloys after 14-day immersion increases. The increase in the total secondary phase creates more micro-galvanic positions, and the shortening phase spacing decreases the distance between the corrosion pits. These two aspects simultaneously promoted the corrosion rate of the ZM-xCa alloys.

Because the SKPFM results show that the Volta potential of the spherical Ca_2_Mg_6_Zn_3_ phase is higher than that of the dendritic one, the spherical phase is electrochemically preferred as a cathode to accelerate Mg matrix (anode) dissolution in the micro-galvanic couple.

**Figure 10:**
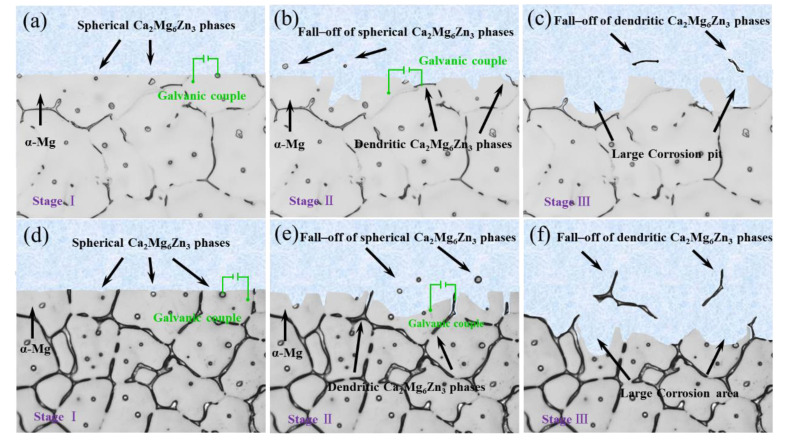
Schematic illustration of corrosion process of the investigated alloys in Hank’s solution: (**a**–**c**) ZM-0.1Ca alloy and (**d**–**f**) ZM-1.0Ca alloy.

We discussed the ion exchange process and formation of the corrosion products during the corrosion process of Mg alloy in our previous studies [3,31]. We do not discuss this in the present investigation. The present research focuses on the effect of secondary phase characteristics on the corrosion behavior of Mg alloys. Figure 10 intuitively illustrates a schematic view of the corrosion behavior of the ZM-0.1Ca and ZM-1.0 alloys. The corrosion process occurs preferentially around spherical phases in stage I (as shown in Figure 10a,d). Because of the dissolution of the Mg matrix around the spherical phase, it falls off and forms corrosion pits in stage II. If the phase spacing is large, for example, in the ZM-0.1Ca alloy, the corrosion process is difficult to proceed to another phase; hence, small corrosion pits are formed (as shown in Figure 10b). As immersion continues, the corrosion process proceeds into grains, and the corrosion process occurs around the dendritic phase distributing at grain boundaries. This corrosion is different from that in grains. Instead of forming corrosion pits, a corrosion “channel” along the dendritic phase is formed, which induces the corrosion process to proceed into the interior grains, as shown in Figure 10b,d. For the alloy with a high Ca content, corrosion penetrates speedily and continuously along grain boundaries. Because of falling off these dendritic phases and dissolution of grains, large corrosion cavities are formed in stage III (see Figure 10f), resulting in a rapid corrosion rate. However, in the case of Zn-0.1Ca alloy, the corrosion pits caused by pitting corrosion are difficult to connect; hence, independent small-scale corrosion pits are generated in the Zn-0.1Ca alloy (as shown in Figure 10c), resulting in slow mass loss.

## 5. Conclusions

1. The cast ZM-xCa (0.1, 0.3, 0.5 and 1.0 wt.%) alloys contain the intragranular spherical Ca_2_Mg_6_Zn_3_ phase and the dendritic one at grain boundaries. The volume fraction of the Ca_2_Mg_6_Zn_3_ phase gradually increased from 2.5% to 7.6%, while its spacing declined monotonically from 43.0 μm to 30.0 μm with an increase in the added Ca amount from 0.1 wt.% to 1.0 wt.%.
2. The Volta potential of the spherical Ca_2_Mg_6_Zn_3_ phase (+109 mV) was higher than that of the dendritic phase (+80 mV); hence, the spherical phase was electrochemically preferred as a cathode to accelerate Mg matrix (anode) dissolution in the micro-galvanic couple.
3. The corrosion rate obtained by weight loss increased slightly with increasing Ca content from 0.1 wt.% to 0.5 wt.% because of the enhanced micro-galvanic corrosion effect. The decrease in the phase spacing and sharp increase in the secondary phase content resulted in a dramatic increase in the corrosion rate of the ZM-1.0Ca alloy.

## Figures and Tables

**Table 1:** Actual compositions of the ZM-xCa alloys (wt.%).

Alloys	Actual Compositions (wt.%)				
	Zn	Mn	Ca	Si	Mg
ZM-0.1Ca	4.13	0.20	0.12	<0.02	Bal.
ZM-0.3Ca	3.81	0.17	0.34	<0.02	Bal.
ZM-0.5Ca	3.78	0.18	0.56	<0.03	Bal.
ZM-1.0Ca	3.76	0.18	0.95	<0.02	Bal.

**Table 2:** Microstructure characteristics of the ZM-xCa alloys (wt.%).

Alloys	Volume Fraction of Secondary Phase (%)	Secondary Phase Spacing (μm)
ZM-0.1Ca	2.5 ± 0.2	43.4 ± 2.0
ZM-0.3Ca	3.0 ± 0.2	39.3 ± 2.1
ZM-0.5Ca	4.2 ± 0.3	32.5 ± 1.5
ZM-1.0Ca	7.6 ± 0.4	30.1 ± 1.2

**Table 3:** Electrochemical parameters of the ZM-xCa alloys.

Alloys	*E_corr_* (V)	*i_corr_* (A/cm^2^)	*E_b_* (V)
ZM-0.1Ca	−1.530	5.90 × 10^−7^	−1.450
ZM-0.3Ca	−1.575	6.63 × 10^−7^	−1.524
ZM-0.5Ca	−1.617	6.77 × 10^−7^	−1.527
ZM-1.0Ca	−1.662	1.31 × 10^−6^	−1.542

**Table 4:** The fitting results of the EIS spectra of ZM-xCa alloys.

Alloys	*R_s_* (Ω cm^2^)	*R_f_* (Ω cm^2^)	*CPE_1_* (10^–6^ F/cm^2^)	n_1_	*CPE_2_* (10^–6^ F/cm^2^)	n_2_	*R_ct_* (kΩ cm^2^)	*R_L_* (kΩ cm^2^)	*L* (H cm^2^)	*R_P_* (kΩ cm^2^)
ZM-0.1Ca	25.48	281.31	7.60	0.73	6.31	0.90	31.29	53.97	2.26 × 10^6^	20.11
ZM-0.3Ca	22.41	278.43	9.13	0.70	6.66	0.89	25.17	70.05	1.61 × 10^6^	18.80
ZM-0.5Ca	22.40	230.21	11.71	0.69	8.83	0.85	45.69	20.25	1.12 × 10^6^	14.28
ZM-1.0Ca	21.57	206.30	19.08	0.66	5.16	0.88	10.80	16.90	2.30 × 10^6^	6.86

## Data Availability

All data are available within the manuscript.
